# Distribution of Molecules Related to Neurotransmission in the Nervous System of the Mussel *Crenomytilus grayanus*

**DOI:** 10.3389/fnana.2020.00035

**Published:** 2020-06-30

**Authors:** Elena Kotsyuba, Alexander Kalachev, Polina Kameneva, Vyacheslav Dyachuk

**Affiliations:** ^1^A.V. Zhirmunsky National Scientific Center of Marine Biology, Far Eastern Branch, Russian Academy of Sciences, Vladivostok, Russia; ^2^Department of Nanophotonics and Metamaterials, ITMO University, St. Petersburg, Russia

**Keywords:** serotonin, neuropeptides, CNS, Bivalves (mussels), ganglia

## Abstract

In bivalves neurotransmitters are involved in a variety of behaviors, but their diversity and distribution in the nervous system of these organisms remains somewhat unclear. Here, we first examined immunohistochemically the distributions of neurons containing different neurotransmitters, neuropeptides, and related enzymes, as well as the proliferative status of neurons in the ganglia of the mussel *Crenomytilus grayanus*. H-Phe-Met-Arg-Phe-NH2 (FMRFamide), choline acetyltransferase (ChAT), γ-aminobutyric acid (GABA) and tyrosine hydroxylase (TH) were found to be expressed by neurons in all the ganglia, whereas serotonin (5-HT) neurons were found only in the cerebropleural and pedal, but not visceral ganglia. Moreover, incubation of living mussels in the presence of a 5-HT precursor (5-HTP) confirmed the absence of 5-HT-containing neurons from the visceral ganglia, indicating that the “serotonin center” of the visceral nervous system is located in the cerebral ganglia. Furthermore, immunostaining of molecules related to neurotransmission together with α-acetylated tubulin demonstrated that this cytoskeletal protein may be a potential pan-neuronal marker in bivalves. Adult mussel neurons do not proliferate, but a population of proliferating PCNA-LIP cells which do not express any of the neurotransmitters examined, perhaps glia cells, was detected in the ganglia. These novel findings suggest that the nervous system of bivalves contains a broad variety of signal molecules most likely involved in the regulation of different physiological and behavioral processes. In addition, proliferating cells may maintain and renew glial cells and neurons throughout the lives of bivalves.

## Introduction

The mollusk nervous system is an outstanding example of plasticity and adaptive capabilities. In comparison to other invertebrates, mollusks occur in a large variety of species that have evolved independently, including free-living and sedentary forms; shelled (gastropods, bivalves) and naked (some nudibranchs, cephalopods) species; and mussels with encephalized nervous systems (Wanninger, 2015). These many different species reflect adaptations to different environments (Bullock and Horridge, 1965), resulting in the performance of different functions by homologous ganglia and nerve networks in closely related taxa. The question of how homologous genes in these different organisms can organize the nervous system in so many different ways remains unanswered.

Anatomically, the organization of the nervous systems of adult bivalves is relatively simply, in part because of their sedentary (attached) lifestyle (Meechonkit et al., 2010; Crook and Walters, 2011). This system consists of three major, bilaterally symmetric pairs of (cerebral, pedal, and visceral) or fused (epiathroid) ganglia. In bivalves, the paired cerebropleural ganglia are connected to one another by commissures, as well as to the fused pedal ganglia via the cerebral-pleural-pedal connectives and to the paired visceral ganglia via long cerebral-pleural-visceral connectives. Cerebropleural ganglia innervate the labial palps, anterior adductor muscle, anterior part of the mantle (via anterior pallial nerves), and sensory organs. The pedal ganglion, which innervates the foot, is absent from species with foot reduction (oysters). A large pair of visceral ganglia innervate the gills (via branchial nerves), heart, posterior adductor muscle, posterior part of the mantle (via posterior pallial nerves) and siphons (Bullough, 1950; Galtsoff, 1964; Grizel et al., 2003; Beninger and Le Pennec, 2006; Gosling, 2015).

The neuromorphology of individual bivalve ganglia has been examined employing classical histochemical approaches (Bullough, 1950; Galtsoff, 1964) and, later, the distributions of individual neurotransmitters in separate mollusk ganglia have been determined immunohistochemically (De Biasi et al., 1984; Karhunen et al., 1993; Too and Croll, 1995; Garnerot et al., 2006; Meechonkit et al., 2010; Tantiwisawaruji et al., 2014). However, the types of neurons in these ganlia and their combined or individual expression of neurotransmitters remain largely unknown.

To understand the functioning of neuronal cells in bivalves, it is essential to know the localization, density, plasticity and nature of the neurotransmitters in the neurons within the ganglia. Therefore, our first goal here was to apply histochemical and immunohistochemical procedures to characterize the diverse localizations of neurotransmitters and peptides, as well as neuronal tubulin, in the neurons of adult *Crenomytilus grayanus* ganglia. The second aim was to identify a reliable marker for proliferating neurons in mussel ganglia.

## Materials and Methods

### Animals

Fifty adult *Crenomytilus grayanus* were collected in July–August 2019 at the Vostok marine station of the A.V. Zhirmunsky National Scientific Center of Marine Biology (42°53′36.8″N, 132°44′03.1″E,10-m depth), an estuary on the inner part of the Vostok Bay and the Peter the Great Bay of the Sea of Japan. These animals were maintained in running seawater with aeration before performing experiments with those 10–15 cm long, 5–6 cm wide, and 4–5 cm thick.

### Dissection

The animals were anesthetized by injecting artificial sterile seawater (ASW) containing 7% MgCI_2_ into their body cavity. The central ganglia were dissected out by cutting the main nerve roots, transferred to 6-well plates, washed twice with ASW (10°C) and then fixed with dissecting needles. Surrounding connective tissue was removed manually using fine forceps and scissors.

### Immunohistochemistry

Dissected ganglia were fixed in 4% paraformaldehyde (PFA) in phosphate-buffered saline (PBS) (pH 7.4) at 4°C for 2–3 h. These samples were subsequently washed in PBS at 4°C for 1 h and cryoprotected by incubation at 4°C overnight in PBS containing 30% sucrose. Neuronal tissues were then embedded in optimal cutting temperature (OCT) medium and frozen at −20°C. Tissues were cut into 4–10 representative 14-μm sections, which were mounted onto slides.

Immunohistochemical analysis was performed on freshly frozen sections, as described previously (Vekhova et al., 2012; Dyachuk et al., 2015). To eliminate non-specific binding, the samples were incubated in blocking buffer [10% normal donkey serum (Jackson ImmunoResearch), 1% Triton-X 100, and 1% bovine serum albumin (Sigma) in 1x PBS] overnight at 4°C, and the primary antibodies were diluted in this same blocking buffer.

The primary and secondary antibodies utilized are described in Table 1.

**TABLE 1:** The primary and secondary antibodies used.

Antibody	Host species	Source, Cat.	Dilution before use
acetylated α-tubulin	Mouse monoclonal	Santa Cruz, sc-23950	1:2000
5-HT	Rabbit polyclonal	Immunostar, 20080	1:2000
5-HT	Goat polyclonal	Immunostar, 20079	1:1000
5-HTP	Rabbit polyclonal	Immunostar, #24446	1:1000
FMRFamide	Rabbit polyclonal	Immunostar, 20091	1:2000
TH	Rabbit polyclonal	EDM Millipore, AB152	1:1000
ChAT (choline acetyltransferase)	Goat polyclonal	EDM Millipore, AB144P	1:500
ChAT (choline acetyltransferase)	Rabbit polyclonal	EDM Millipore, AB 143	1:500
GABA	Rabbit polyclonal	Sigma-Aldrich, A2052	1:500
PCNA	Mouse polyclonal	Santa Cruz Biotechnology, sc-56	1:200
Alexa Fluor 555 anti-goat	Donkey	Life Technologies, A21432	1:1000
Alexa Fluor 488 anti-rabbit	Donkey	Life Technologies, A21206	1:1000
Alexa Fluor 555 anti-rabbit	Donkey	Life Technologies, A31572	1:1000
Alexa Fluor 647 anti-mouse	Donkey	Life Technologies, A31571	1:1000
Alexa Fluor 555 anti-mouse	Donkey	Life Technologies, A31570	1:1000

For double immunostaining, antibodies raised in different animals were used in combination: sequential addition of the primary and secondary antibodies for the first antigen and thereafter for the second and third antigens, with monitoring of immunostaining by confocal microscopy following each step. The mixtures of primary antibodies were either anti-goat 5-HT with anti-rabbit FMRFamide or GABA or TH, or anti-rabbit 5-HT with anti-goat ChAT. The secondary antibody mixtures consisted of donkey anti-goat (DAG) 555 with donkey anti-rabbit (DAR) 488 or DAG-488 with DAR-555 (all from Thermo Fisher Scientific). Immunostaining was also performed with anti-rabbit FMRFamide + anti-goat 5-HT or anti-rabbit 5-HTP + anti-goat 5-HT and the corresponding secondary antibodies DAR-488 + DAG-555. In other cases anti-α-acetylated mouse tubulin or anti-mouse PCNA were mixed with anti-rabbit 5-HT, utilizing donkey anti-mouse (DAM)-488 with DAR-555 secondary antibodies.

All primary antibodies were first incubated with tissue sections overnight at 4°C, followed by triple rinses with PBS containing 0.1% Tween-20. Subsequently, the slides were incubated with the secondary antibodies for 2 h at room temperature. The mixtures of secondary antibodies always contained 4′,6-diamidino-2-phenylindole (DAPI, Sigma-Aldrich) as well. The slices were then mounted with glycerol-based media (Merck & Co., Kenilworth, NJ, United States). As a control for non-specific immunorecognition, we performed immunohistochemical staining without the primary antibodies, adding only the secondary antibodies or normal (non-immunized) immunoglobulin G (1:500-1:1000; Sigma-Aldrich; I5006, I5381, and I5256).

### Formaldehyde-Glutaraldehyde-Induced Fluorescence (FaGlu)

To bypass the problem of non-functional antibodies against dopamine, formaldehyde-glutaraldehyde-induced fluorescence technique (FaGlu) was employed for the detection of catecholamines, as described earlier (Smith, 1982), with appropriate modification for cryosections. The CPG, PG, or VG was fixed for 4 h at room temperature in 4% PFA/0.5% glutaraldehyde and 30% sucrose in 0.1 M PBS (pH 7.4) and then rinsed with 0.1 M PBS, air-dried overnight, and mounted between glass coverslips using Mowiol medium (Calbiochem, San Diego, CA, United States).

### Treatment of Bivalves With 5-HT and 5-HTP

Adult *Crenomytilus grayanus* were maintained in the presence of 5-HT (10^–7^ M) and 5-HTP (10^–5^ M) with ascorbic acid for 2 h at 15°C. Subsequently, these animals and untreated controls were rinsed with fresh seawater and dissected in sterile ASW containing 7% MgCI_2_, following which the visceral ganglia were cryosectioned and fixed in 4% PFA in PBS for immunohistochemical detection of neurons containing 5-HT-LIP.

### Microscopy and Imaging

All images were acquired using a Zeiss LSM 780 confocal microscope (Carl Zeiss) and processed and analyzed with the Imaris (Bitplane, Zurich, Switzerland) and Image J (National Institutes of Health, Bethesda, MD, United States) software, the latter also being used for three-dimensional visualization and analysis of confocal stacks.

FaGlu fluorescence was examined under a Zeiss LSM 780 laser scanning microscope (Carl Zeiss, Oberkochen, Germany), operated in the λ-mode. The excitation wave length was 405 nm and the emission signal registered at 32 evenly spaced wavelengths (8.9 nm apart) from 408–693 nm utilizing a QUASAR detector [the peak of dopamine fluorescence is at 485–500 nm (Furness et al., 1977)]. The results obtained were unmixed linearly with the Zeiss Zen 2.1 SP3 (Black Edition) software.

### Quantification and Statistical Analysis

Consecutive series of longitudinal and transverse sections of mussel tissue were examined. The numbers of cells expressing specific molecular markers were counted in 10–20 representative sections in each of two whole CPGs, one fused PG and the VGs of at least three mussels (biological *n* = 3–6). Only cells with a visible nucleus were counted. All quantification was performed with the Image J software, processed in the Prism 7 software (GraphPad, San Diego, CA, United States) and presented as means ± standard errors of the mean for each ganglion and type of neuron.

### Ethical Considerations

The mussels studied are not an endangered or rare invertebrate species. Access to the marine area, which is owned by the Russian state, did not require any special permission.

## Results

### General Morphology of Nervous System

**FIGURE 1:**
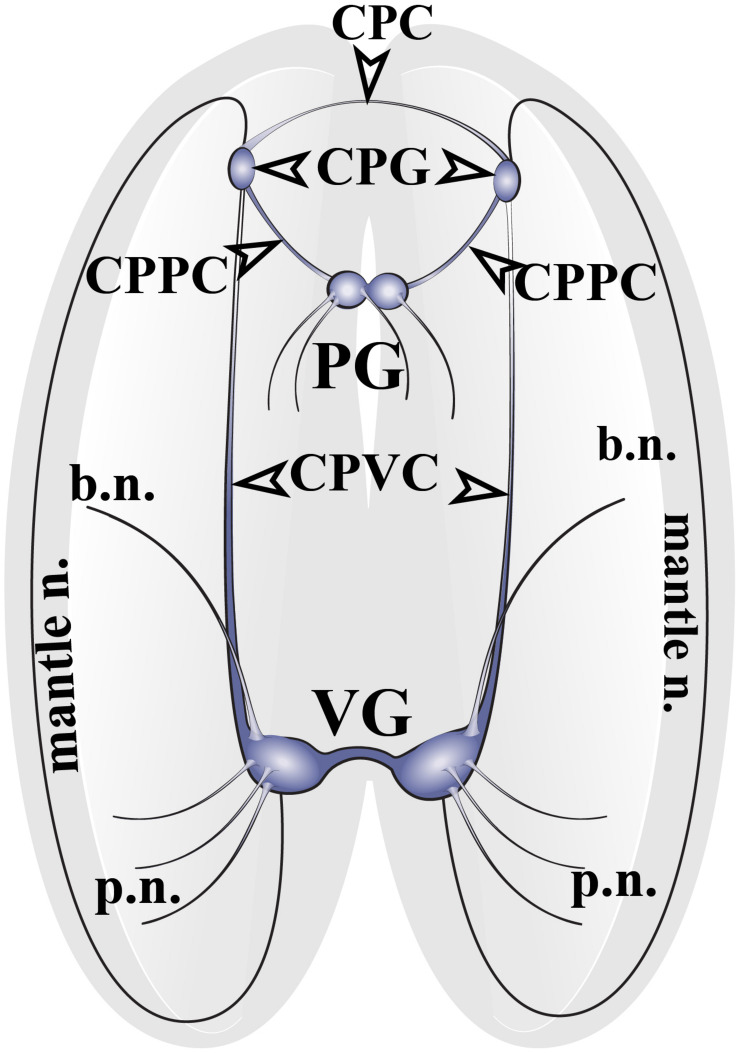
The structure of the nervous system of the mussel *Crenomytilus grayanus*. The nervous system of this mussel consists of paired cerebropleural ganglia (CPG) connected to one another by cerebropleural commissures (CPC) and to the pedal ganglia (PG) via the cerebral-pleural-pedal connectives (CPPC) and to the paired visceral ganglia (VG) via long cerebral-pleural visceral connectives (CPVC). The paired visceral ganglia are connected by to one another by a thick, short visceral commissure. The CPG and VG innervate the anterior and posterior regions of the mantle via pallial (p.n.) and mantle nerves (mantle n.). VG innervates the gills via branchial nerves (b.n.).

**FIGURE 2:**
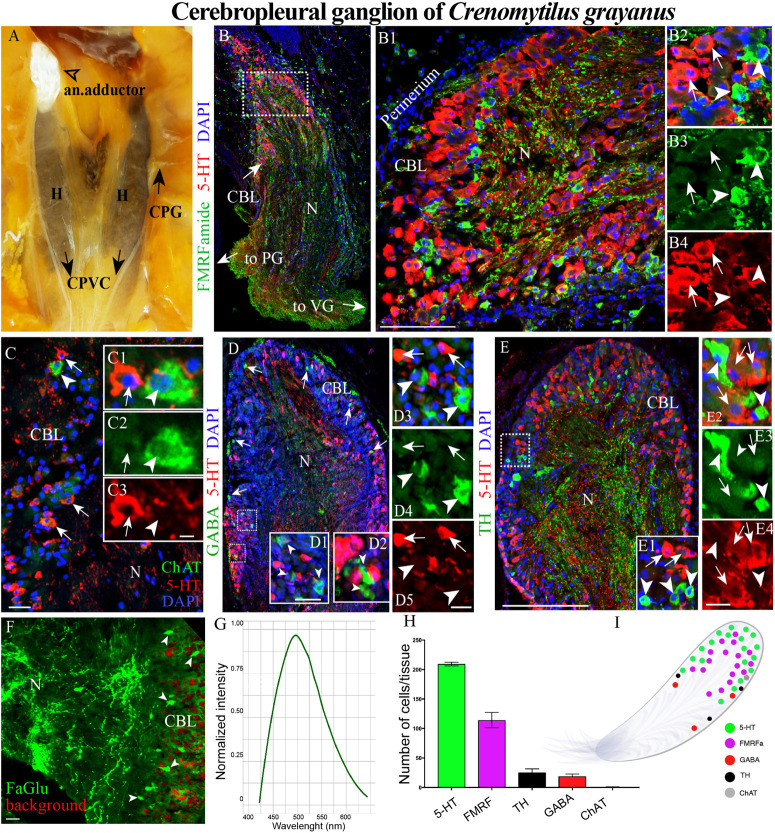
The morphology of the cerebropleural ganglia (CPG) and its neurotransmitter heterogeneity **(A)** The CPG in the living body of a mussel. **(B)** The distribution of 5-HT- and FMRFamide-LIP in the cell body layer (CBL) of the CPG. Arrows indicate the connectives joining the CPG with the pedal (PG) and visceral ganglia (VG). **(B1)** 5-HT- and FMRFamide-LIP neurons in the CBL of the CPG. **(B2–B4)** Split channels, arrows indicate 5-HT-LIP neurons, arrowheads FMRFamide-LIP neurons. **(C)** Immunodetection of ChAT-LIP neurons (arrowhead) and simultaneous visualization of 5HT-LIP neurons (arrows). **(C1–C3)** Split channels. **(D)** Double immunostaining for GABA (arrow heads) and 5-HT (arrows). **(D1,D2)** The magnified portion demonstes the absence of 5-HT expression in GABA-LIP neurons in the cell body layer. **(D3–D5)** Split channels. **(E,E1)** Simultaneous visualization of TH (arrowheads) and 5-HT (arrows). **(E2–E4)** Split channels. **(F)** Formaldehyde/glutaraldehyde-based histochemical detection of dopaminergic neurons (arrow heads, green color) in the CPG. Red is simply the background color. **(G)** Emission spectrum of dopamine after λ- scanning. **(H)** Quantitative analysis of cells with immunopositive reaction in the CPG. **(I)** Schematic representation of the distribution of neurons expressing different neurotransmitters. 5-HT, 5-hydroxytryptamine; ChAT, choline acetyltransferase; TH, tyrosine hydroxylase; N, neuropil; an. adductor, anterior adductor; H, hepatopancreas; CPVC, cerebral-pleural-visceral connectives; Scale bars: **(E)**, 1 mm; **(B1)**, 100 μm; **(C)**, 20 μm; **(C3,D1,D5,E4,F)** 10 μm.

**FIGURE 3:**
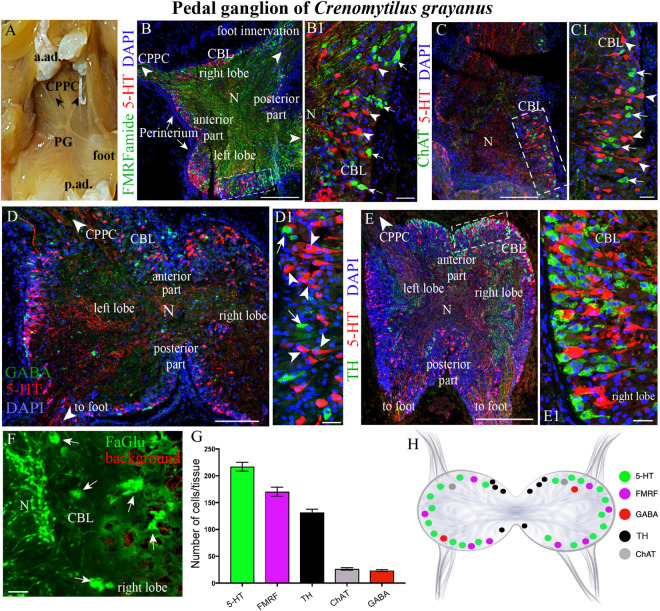
The morphology of the pedal ganglia (PD) and its neurotransmitter heterogeneity. **(A)** View of the PG in an adult mussel body. **(B,B1)** The distribution of 5-HT-(arrowheads) and FMRFamide-LIP (arrows) neurons in the cell body layer (CBL) of the PG. **(C,C1)** Localization of 5-HT (arrowheads) and ChAT-immunoreactivity (arrows). **(D,D1)** Lack of colocalization of GABA with 5-HT-LIP neurons in the CBL of the PG. **(E,E1)** Simultaneous visualization of TH and 5-HT. **(F)** Formaldehyde/glutaraldehyde-based histochemical detection of dopaminergic neurons (arrows, green color) in the PG. Red is simply the background color. **(G)** Quantitative analysis of neurons with different neurotransmitters. **(H)** Schematic representation of the distribution of neurons in the PG. 5-HT, 5-hydroxytryptamine; GABA, γ-aminobutyric acid; TH, tyrosine hydroxylase; CPPC, cerebral-pleural-pedal connectives; a. ad., anterior adductor; p. ad., posterior adductor; N, neuropil. Scale bars: **(B–E)**, 1 mm; **(B1,C1,D1,E1,F)**, 20 μm.

**FIGURE 4:**
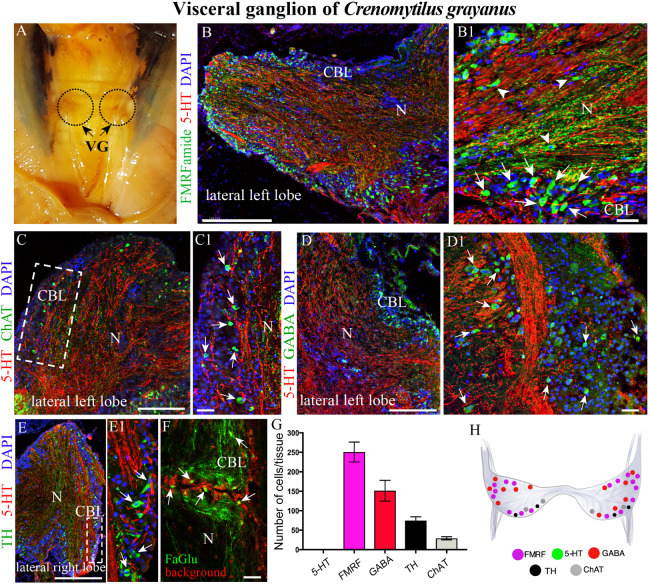
The morphology of the visceral ganglia (VG) and its neurotransmitter heterogeneity. **(A)** View of the paired VG in an adult mussel. **(B,B1)** Immunostaining for 5-HT and FMRFamide in the cell body layer (CBL) of the VG. The arrows indicate FMRFamide-LIP neurons, some of which are located in the neuropile (arrowheads). Note the absence of 5-HT-LIP neuronal somata. **(C,C1)** Double immunostaining for 5-HT and ChAT (arrows) shows no colocalization. **(D,D1)** The distribution of GABA (arrows) and 5-HT. **(E,E1)** Simultaneous visualization of TH (arrows) and 5-HT. **(F)** Formaldehyde/glutaraldehyde-based histochemical detection of dopaminergic neurons (arrows, green color) in the VG. Red is simply the background color. **(G)** Quantitative analysis of neurons with different neurotransmitters. **(H)** Diagram of the VG showing the distribution of the neurotransmitters examined. 5-HT, 5-hydroxytryptamine; ChAT, choline acetyltransferase; GABA, γ-aminobutyric acid; TH, tyrosine hydroxylase; N, neuropil. Scale bars: **(B–E)**, 0.5 mm; **(B1,C1,D1,E1,F)**, 25 μm.

The nervous system of adult *Cr. grayanus* consists of paired cerebropleural ganglia (CPG), a fused pedal ganglion (PG) and paired visceral ganglia (VG), (Figure 1 and Supplementary Figures S1A–C). The CPG are joined by a cerebral commissure; the PGs are fused ganglia; and the VGs are connected to one another by short and thick visceral commisures (Supplementary Figures S1A–C). All ganglia are linked by cerebropleuropedal and cerebral-pleural-visceral connectives (Figure 1). All *Crenomytilus* ganglia have common structural features: they are covered by a protective sheath, the perineurium; and they consist of two parts: the cell body layer (CBL), where all the neuronal somata are located, and the neuropil (N) formed by cell processes, which are concentrated in the center of the ganglia and radiate outward (Figures 2–4 and Supplementary Figure S1).

### Immunohistochemical Detection of Neuronal Elements in the Cerebropleural Ganglion

Among the *Cr. grayanus* ganglia, the CPGs are the smallest paired ganglia (each 1 ± 0.4 mm long, 0.7 ± 0.35-mm wide and 0.6 ± 0.27 mm thick), with approximately 17000 ± 119 cells per ganglion (Figures 2A,B and Supplementary Figure S1A). In living adults, this minuscule pair of ganglia are present as elongated bodies with a thickened base (Figures 2A,B and Supplementary Figure S1A). 5-Hydroxytryptamine- (5-HT) and FMRFamide-like immunopositive (hereafter abbreviated as –LIP) neurons were detected in the cell body layer (Figures 2B–B4, Supplementary Figures S1A, and Supplementary Videos S1, S2), most 5-HT-LIP neurons (15–25 μm) in the outer part and small FMRFamide-LIP neurons (7–12 μm) in deeper parts (Figure 2B1 and Supplementary Figure S1A). None of the 5-HT-LIP neurons were found to express FMRFamide (Figures 2B2–B4).

Only 1 or 2 ChAT-LIP neurons were present in each section from the cell body layer of the CPG (Figures 2C–C3). γ-Aminobutyric acid (GABA) was expressed in the cell body layer and to some extent in the neuropile (Figures 2D,D1–D5). All of the tyrosine hydroxylase- (TH)-LIP neurons were observed in the cell body layer (Figures 2E–E4). Thus, the cell body layer of the CPG contains 5- HT-, FMRFamide-, ChAT-, GABA-, and TH-LIP neurons, each of which expresses only a single transmitter, with no colocalization.

The formaldehyde-glutaraldehyde (FaGlu) fluorescence reaction commonly employed to detect catecholamines (Furness et al., 1977) is especially useful in the absence of working antibodies against dopamine. Application of this reaction revealed the presence of a set of catecholamine-containing cells in the CPG and λ-mode scanning showed that this was dopamine, with a peak signal at 495–500 nm (Figures 2F,G).

Of the total immunopositive cells in the CPG, 56 + 1.6% (207 ± 6 cells) were 5-HT-LIP, 31 + 1.3% (114 ± 5) FMRFamide-LIP, 7 + 0.2% (26 ± 1) TH-LIP, 5.1 ± 0.2% (19 + 1) GABA-LIP and 0.2 ± 0.2% (1 + 1) ChAT (Figure 2H). In summary, the CPG consists of several types of neurons each expressing only one of several different signal molecules, with 5-HT and FMRFamide being the predominant neurotransmitters (Figure 2I).

### Immunohistochemical Detection of Neuronal Elements in the Pedal Ganglia

The fused PG is located in the visceral mass at the base of the foot (Figure 3A and Supplementary Figure S1B). In frontal projection, the PGs resemble a butterfly, with nerves emerging from both the anterior and posterior surfaces (Figures 3A,B and Supplementary Figure S1B). This structure is 2 ± 0.5 mm long, 1.0–1.1 ± 0.21 mm wide, and 0.8 ± 0.29 mm thick, containing altogether 39428 ± 257 cells.

As also observed in the case of the CPGs, 5-HT- (15–25 μm) and FMRFamide-LIP (7–12 μm) neurons (Figures 3B–B1, Supplementary Videos S3, S4, and Supplementary Figure S1B), as well as ChAT-LIP (Figures 3C,C1), GABA-LIP (Figures 2D,D1), and TH-LIP (Figures 3E,E1) neurons were all detected in the cell body layer of PG. Again, each of these neurons expresses only a single neurotransmitter, with no colocalization. In addition, dopamine-containing neurons were present in this same cell body layer (Figure 3F).

Of the total immunopositive cells in the PG, 38.1 ± 1% (217 ± 6 cells) were 5-HT-LIP, 29.8 ± 1.5% (170 ± 9) FMRFamide-LIP, and 23.2 ± 0.7% (132 ± 4) TH-LIP, with significantly fewer cells expressing CHAT (4.7 ± 0.3%, 27 ± 2 cells) or GABA (4 ± 0.3%, 23 ± 2) (Figure 3G). In summary, the PG of *Cr. grayanus* contains more cells than the CPG, with the main neuronal populations expressing 5-HT, FMRFamide, and TH (Figure 3H).

### Immunohistochemical Detection of Neuronal Elements in the Visceral Ganglia

The paired VG is the largest ganglion in *Cr. grayanus* (4.4 ± 0.87 mm long, 1.6 ± 0.65 mm wide, 1.2 ± 0.37 mm thick), with approximately 506 neurons per tissue section (61714 ± 5228 cells in total). The paired ganglia of this structure are connected by a thick commissure (Figure 4A and Supplementary Figure S1C).

Surprisingly, the high density of 5-HT-LIP neurons detected in both the CPG and PG was absent from the VG (Figure 4B and Supplementary Figure S1C). Thus, double immunostaining with antibodies against 5-HT and FMRFamide revealed only small FMRFamide-LIP neurons (7–12 μm) in the cell body layer, with other neurons of this kind in the neuropile (Figures 4B,B1 and Supplementary Video S5). High-magnification images of this staining confirmed the absence of 5-HT-LIP neurons, but presence of 5-HT-LIP cell processes that, together with FMRFamide-LIP fibers, formed the neuropil (Figure 4B1). As observed in the cell body layer of the PG, the VG contains ChAT-LIP neurons, located in this case in the external portion of this layer close to the neuropile (Figures 4C,C1).

Unlike the other ganglia, the cell body layer of the VG contained large numbers of GABA-LIP neurons (Figures 4D,D1). Moreover, this same layer contained TH-LIP (Figures 3E,E1) and dopamine-fluorescent neurons (Figure 4F). Thus, the cell body layer of the VG does not contains 5-HT-LIP neuronal somata, but does harbor neurons that express FMRFamide, ChAT, GABA, TH, and dopamine and 5-HT-LIP cell processes in neuropile, again with no colocalization.

Of the total immunopositive cells in the VG, 49.8 ± 3.9% (252 ± 20 cells) were FMRFamide-LIP, 29.8 ± 1.7% (151 ± 9) GABA-LIP, 14.6 ± 0.7% (74 ± 4) TH-LIP, and 5.7 ± 0.3% (29 ± 2) ChAT-LIP (Figure 3G). A distinctive feature of the VG was the absence of 5-HT-LIP neurons, which dominated the CPG and PG (Figure 4H).

**FIGURE 5:**
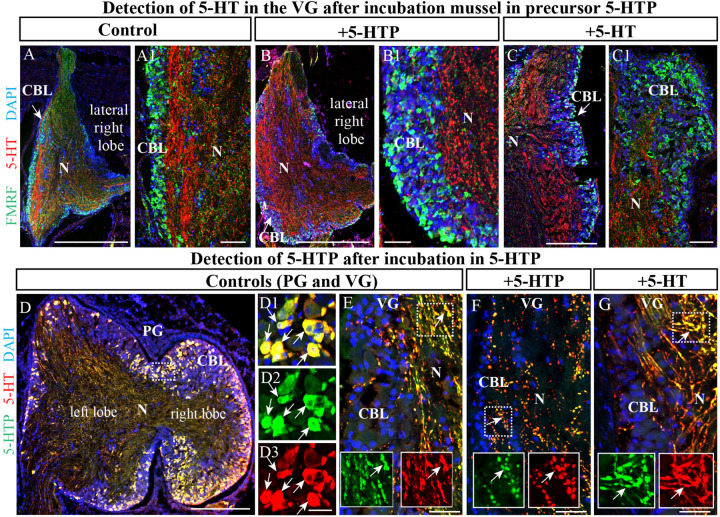
The visceral ganglia (VG) do not contain 5-HT-LIP neurons. **(A)** An untreated control VG (lateral right lobe) after staining with antibodies against FMRFamide and 5-HT. **(A1)** The inset shows a magnified image demonstrating the absence of 5-HT-LIP neuronal somata and the presence of FMRFamide-LIP neurons in the cell body layer. **(B)** A representative view of the VG (lateral right lobe) after exposure to the 5-HT precursor 5-HTP. **(B1)** The inset shows a magnified image demonstrating the absence of 5-HT-LIP neurons. **(C)** The VG after incubation with 5-HT. **(C1)** The magnified portion demonstrates the absence of 5-HT- and FMRFamide-LIP neurons in the cell body layer. **(D)** Simultaneous visualization of 5-HTP and 5-HT in the PG **(D1–D3)** (the arrows indicate cell bodies with co-localization) and VG as positive controls **(E)** with inserts (split channels) and the absence of 5-HT-LIP neurons in the VG after the incubation **(F,G)** (the arrows indicate neurites with colocalization). 5-HT, 5-hydroxytryptamine; 5-HTP, 5-hydroxytryptophan; PG, pedal ganglion; CBL, the cell body layer; N, neuropil. Scale bars: **(A–D)**, 1 mm; **(A1,B1,C1,D1–D3,E–G)**, 50 μm.

To further examine the unexpected observation that the largest mussel ganglion, the VG, which innervates the catch muscle (posterior adductor), heart, and kidney, contains so few 5-HT-LIP neurons, mussels were incubated with the precursor of 5-HT, 5-hydroxytryptophan (5-HTTP), which is converted to 5-HT by 5-HTP-decarboxylase. Even under these conditions, no 5-HT-LIP neurons were detected in this ganglion (Figures 5A,A1,B,B1, 4C,C1). In addition, double immunostaining showed 100% colocalization of 5-HT and 5-HTP in the PG (Figures 5D–D3), but an absence of any immunopositive neurons in the VG (Figures 5E–G). In contrast, the neuropil of all of the ganglia immunostained positively for 5-HT-LIP and 5-HTP-LIP (Figures 5D–G). Therefore, we conclude that the VG does not contain 5-HT-LIP cell bodies or the enzymatic machinery for biosynthesis of 5-HT, but does contain 5-HT-LIP and 5-HTP-LIP fibers.

### Acetylated α-Tubulin Colocalizes With 5-HT and FMRFamide

**FIGURE 6:**
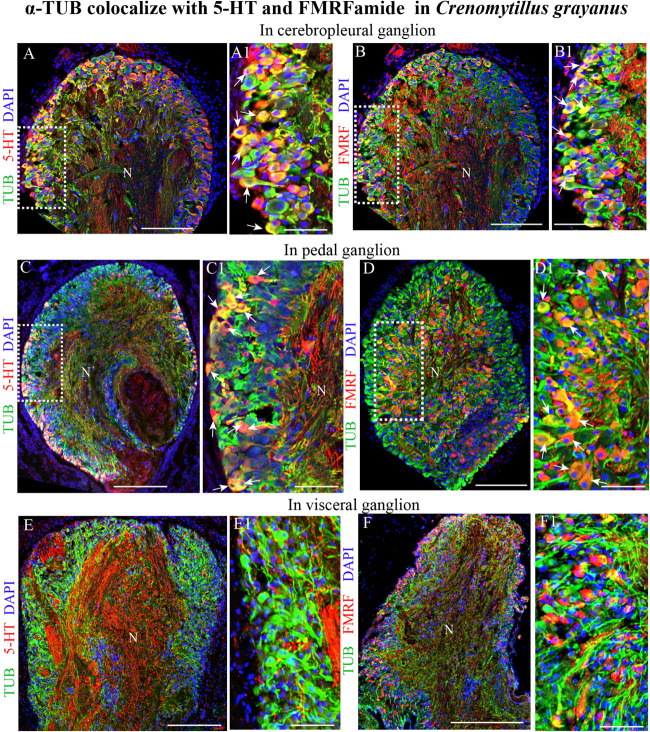
Acetylated α-tubulin (TUB) colocalizes with 5-HT and FMRFamide in the mussel *Crenomytilus grayanus*. **(A,B)** Simultaneous visualization of TUB and 5-HT **(A,A1)** or FMRFamide **(B,B1)** in the CPG. 5-HT and FMRFamide are expressed in all TUB-immunopositive neurons (arrows). **(C,D)** Double-immunostaining for TUB and 5-HT **(C,C1)** or FMRFamide **(D,D1)** in the PG. Double-immunostained neurons indicated by arrows. **(E,F)** Simultaneous visualization of TUB with 5-HT **(E,E1)** or FMRFamide **(F,F1)** in the VG. N, neuropil. Scale bars: **(A–F)**, 100 μm; **(A1,B1,C1,D1,E1,F1)**, 60 μm.

Classical histochemical approaches and bright-field microscopy have been utilized extensively to examine the gross anatomy of the nervous system of bivalves (Galtsoff, 1964; Bullock and Horridge, 1965). However, no single marker for all elements of the nervous system of mollusks is yet commonly recognized (Wanninger, 2015; Schmidt-Rhaesa et al., 2016). After double immunostaining for acetylated α-tubulin in combination with 5-HT or FMRFamide, all tubulin-immunopositive cells in the CPG (Figures 6A–B1) and PG (Figures 6C–D1) were also found to express one of these transmitters (only FMRFamide in the case of the VG) (Figures 6E–F1). This finding indicates that tubulin is a potential candidate for a pan-neuronal marker in bivalves.

### Neurons in Adult *Crenomytilus grayanus* Ganglia Do Not Proliferate

**FIGURE 7:**
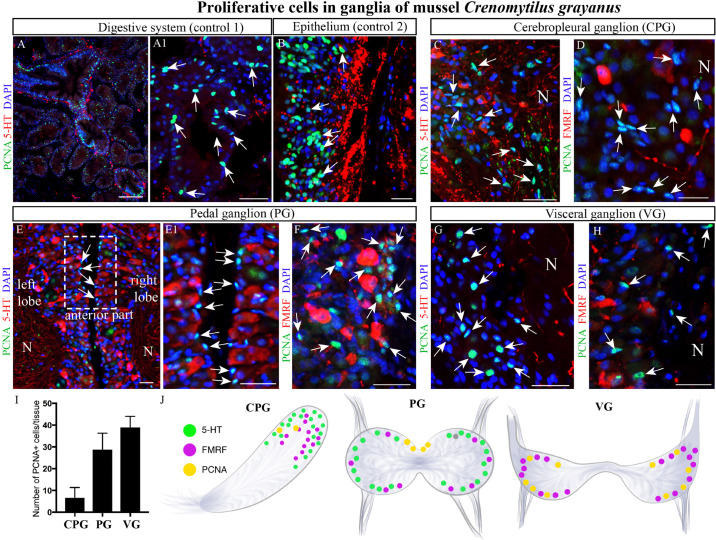
Proliferating cells in ganglia of the mussel *Crenomytilus grayanus.* **(A,B)** PCNA-immunostaining of the digestive system (control 1; **A,A1**) and mantle epithelium (control 2; **B**) as positive controls. PCNA-LIP cells (arrows) among the 5-HT- and FMRFamide-LIP neurons in the CPG **(C,D)**, PG **(E,E1,F)**, and VG **(G,H)**. Note, these PCNA-LIP cells do not express 5HT or FMRFamide. **(I)** Quantitative analysis of PCNA-LIP cells in mussel ganglia. **(J)** schematic illustration summarizing the localization of PCNA-LIP cells in mussel ganglia. PCNA, proliferating cell nuclear antigen; N, neuropil; PG, pedal ganglion; VG, visceral ganglion; CPG, cerebropleural ganglion. Scale bars: **(A)**, 100 μm; **(A1,B–D)**, 50 μm; **(E)**, 15 μm; **(E1–H)**, 20 μm.

Initially, we utilized primary antibodies against Ki67, pH3, and PCNA to detect proliferating cells in these ganglia and found that PCNA is the most specific of these. As positive controls for cell proliferation, the digestive system (Figures 7A,A1) and mantle epithelium (Figure 7B) were both found to contain numerous PCNA-immunopositive cells (Figures 7A,B). Subsequent analysis of the CPG revealed 3–6-μm PCNA-immunopositive cells (8.5 ± 1.17 cells/section) among the most common FMRFamide- and 5-HT-LIP neurons (Figure 7C,D). The cell body layers of the PG and VG also contained small (4–8-μm) PCNA-immunopositive cells (37 ± 2.06 cells and 50.07 ± 1.61 cells/section, respectively, Figure 7I), which were negative for FMRFamide and 5-HT (Figures 7E,E1,F–H,J). Thus, we conclude that the neurons in mussel ganglia do not divide. The proliferating cells detected in the CPG, PG and VG are small and elongated like glia, but their nature remains to be elucidated.

## Discussion

The bivalve nervous system is tetranervous, consisting of a visceral (lateral) portion containing cerebral, pleural, and visceral ganglia located along lateral nerve cords (VNC) and a ventral (pedal) nervous system including pedal ganglia and a paired pedal cord (Yurchenko et al., 2018). Our anatomical findings on the nervous system of the mussel *Crenomytillus grayanus* here are in agreement with the basic organization of the nervous system of other bivalve species (Bullock and Horridge, 1965; De Biasi et al., 1984; Matsutani and Nomura, 1984; De Biasi and Vitellaro-Zuccarello, 1987; Karhunen et al., 1993; Too and Croll, 1995; Siniscalchi et al., 2004; Meechonkit et al., 2010; Tantiwisawaruji et al., 2014). Our current findings suggest that neurons in the nervous system of the mussel *Cr. grayanus* express a broader spectrum of neurotransmitters and neurotransmission-related molecules than previously identified in the marine mussels *Mytilus* (De Biasi and Vitellaro-Zuccarello, 1987; Vitellaro-Zuccarello et al., 1991) and *Mytilus edulis* (Mathieu et al., 1991), a freshwater mussel *Hyriopsis bialata* (Meechonkit et al., 2010), the clams *Venus verrucosa* (Siniscalchi et al., 2004) and *Macoma balthica* (Karhunen et al., 1993), and the scallops *Patinopecten yessoensis*, *Placopecten magellanicus*, and *Pecten* maxi (Matsutani and Nomura, 1986; Karhunen et al., 1993; Too and Croll, 1995). The neurons of *Cr. grayanus* expressing 5-HT, FMRFamide and GABA are strikingly similar in morphology to those of the closely related mussels *Mytilus galloprovincialis* (with respect to those expressing 5-HT and GABA) (Vitellaro-Zuccarello et al., 1991) and *Mytilus edulis* (FMRFamide) (Mathieu et al., 1991). All neurons in the *Cr. grayanus* that express neurotransmitters (with the exception of serotonin-LIP neurons in the visceral ganglia) and other molecules related to neurotransmission are located in the cell body layers (outer cell zones) of the ganglia, with their cell processes forming the neuropil. Interestingly, no neurons expressing more than one transmitter were observed in any ganglia here.

Another interesting observation is the lack of clusters of neurons expressing one and the same transmitter in the *Cr. grayanus*. In other bivalves clusters of neurons at specific sites in the cell body layer of ganglia express the same transmitter, for example, ganglionic 5- HT-, FMRFamide-, histamine-, and GABA-LIP neurons in the scallops *Placopecten magellanicus* (Too and Croll, 1995) and *Patinopecten yessoensis* (Matsutani and Nomura, 1984), 5-HT-LIP neurons in the clam *Venus verrucosa* (Siniscalchi et al., 2004), and monoamine- and histamine-LIP neurons in *Macoma* (Karhunen and Panula, 1991; Karhunen et al., 1993). It is noteworthy that such neuronal clusterization is detected only in bivalves that move freely and exhibit complicated behavior [e.g., scallops that can swim by spasmodic clapping movements of their valves or clams that control their movement with their foot (digging)], not in attached mussels and oysters. Therefore, we propose that this difference may reflect the complexity of neuromuscular circuits. Such grouping of neurons is well-known in gastropods (Vallejo et al., 2014; Acker et al., 2019) and cephalopods (Wollesen et al., 2010; Webber et al., 2017; Shigeno et al., 2018) on land or in water, animals that have developed appropriate sensory-motor neuronal machinery.

The distributions of neuroactive substances (transmitters, modulators, and peptides) in the nervous systems of several mollusks have been characterized by immunohistochemical (De Biasi and Vitellaro-Zuccarello, 1987; Karhunen et al., 1993; Siniscalchi et al., 2004; Garnerot et al., 2006; Meechonkit et al., 2010) and histochemical procedures (formaldehyde- or glyoxylic acid-based visualization of monoamines) (Welsh, 1957; Furness et al., 1977; Smith, 1982; Wreford et al., 1982; Croll and Chiasson, 1990), as well as by chromatography (Welsh, 1957; Smith, 1982) and pharmacological/electrophysiological approaches (Yojiro et al., 1991; Yuko et al., 1991). We found that both serotonin and FMRFamide are expressed at very high levels in the nervous system of *Cr. grayanus*, in 49.8% of the total number of neurons in the visceral ganglia up to 87% in the cerebropleural ganglia. Moreover, the nervous system of bivalves contains much higher 5-HT concentrations than that of other invertebrates (Alavi et al., 2017).

**FIGURE 8:**
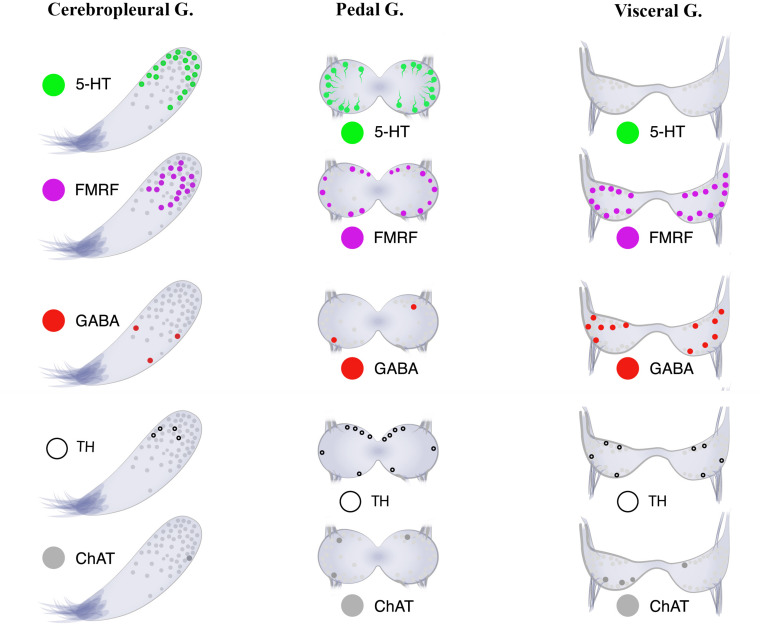
Neurotransmitter atlas: The neurotransmitter diversity of neurons in the three ganglia [cerebropleural (CPG), pedal (PG), and visceral (VG) ganglia] of the mussel *Crenomytilus grayanus.*

The absence of 5-HT-LIP cells in the visceral ganglia of *Cr. grayanus* (Figure 8), even after incubation with a serotonin precursor (5-HTP), was unexpected. 5-HT-LIP neurons have been detected in the visceral ganglia of the free-living clam *Venus verrucosa* (Siniscalchi et al., 2004) and only in the accessory portion of the visceral ganglia of the scallop *Patinopecten yessoensis*, but are absent from attached mussels and oysters (Kotsyuba, unpublished data).

Despite this absence of 5-HT-LIP somata, numerous processes in the neuropil of the visceral ganglia, possibly originating from the cerebropleural ganglia via cerebral-pleural-visceral connectives and/or peripheral neurons, do demonstrate such immunostaining. In support of the ganglionic origin of visceral 5-HT-LIP processes, rapid axonal transport of radioactively labeled amino acids via the long cerebral-visceral connectives toward the VG has been observed in the freshwater mussel *Anodonta cygnea* (McLaughlin and Howes, 1973). Moreover, following injection of ^3^H-5-HTP into the cerebral ganglia of *Anodonta*, radiolabeling was detected in the cerebral-visceral connective (Howes et al., 1974). At the same time, the potential peripheral origin of 5-HT-LIP processes in the VG neuropile remains unexplored and cannot be excluded. It seems even less likely that the 5-HT-LIP processes that extend from the neuronal cell bodies of the pedal ganglion pass through the cerebral ganglion into the visceral ganglia.

Thus, the 5-HT-LIP somata whose processes pass through the VG probably remain in the CPG and are involved in the innervation of visceral organs, such as the gonads, gills, hearts, sensory organs, posterior adductor muscle, and parts of the mantle (Galtsoff, 1964; Beninger and Le Pennec, 2006). Although in experiments physiological concentrations of 5-HT exerted a pacemaking effect on bivalve mollusks (Kuwasawa and Hill, 1997), in some species this compound has inhibitory effects on the heart (Greenberg and Price, 1980). Moreover, 5-HT regulates contraction and relaxation of the anterior byssus retractor muscle (ABRM) in the blue mussel *Mytilus edulis* (Twarog, 1954) and *Mytilus galloprovincialis* (Vitellaro-Zuccarello et al., 1990), in agreement with pharmacological and physiological evidence for a role of serotonin in the control of catch contraction (Muneoka and Twarog, 1983). In addition, immunohistochemical (Vitellaro-Zuccarello et al., 1990) and physiological findings (Aiello et al., 1981) indicate that in *Mytilus edulis*, the intrinsic foot and extrinsic posterior retractor muscles are innervated by serotoninergic neurons.

Actually, we found that approximately 56% of the cells in the CPGs of *Cr. grayanus* are 5-HT-LIP neurons (the “serotonic center” of the visceral nervous system consisting of the CPG, VG and their nerve connections), in agreement with previous observations that in the clams *Tresus capax* and *Macoma nasuta* (Smith, 1982; Alavi et al., 2017) the level of 5-HT is higher in the CPG than the VG. The 5-HT content of bivalve ganglia is influenced by the reproductive cycle, seasonal physiological activities, and various stress factors (Fingerman and Nagabhushanam, 1997). This neurotransmitter is also involved in innervation of the gonad, gamete maturation, and germ cell emission (Matsutani and Nomura, 1982; Hirai et al., 1988; Deguchi and Osanai, 1995; Alavi et al., 2014). In this connection, serotonin is actually used to stimulate spawning in mollusks (Dyachuk, 2013; Yurchenko et al., 2018, 2019). The transmitter expressed at the next highest level in the bivalve nervous system is FMRFamide. FMRFamide-LIP neurons were found here in all of the ganglia in *Cr. grayanus* (Figure 8) and have been described in the cerebral, pedal, and parietovisceral ganglia of the scallops *Placopecten magellanicus* (Too and Croll, 1995) and *Pecten maximus* (Henry et al., 1995), the stellate ganglia of the squid *Loligo pealei* (Burbach et al., 2014), and the abdominal ganglion of gastropods (Voronezhskaya and Croll, 2015). In *Cr. grayanus* FMRFamide-LIP neurons are located primarily in the cell body layers, but some are also found in the neuropiles of all ganglia. Indeed, FMRFamide-LIP processes form the neuropil of all ganglia. Moreover, the FMRFamide-LIP cells are smaller (7–12 μm) than 5-HT-LIP cells (15–20 μm). Finally, FMRFamide-LIP neurons also immunostained for acetylated α-tubulin, but never for other neurotransmitters or other molecules related to neurotransmission. These findings indicate that acetylated α-tubulin is expressed by all neurons in bivalves and may serve as a pan-neuronal marker in these animals.

FMRFamide was first discovered in the clam *Macrocallista nimbosa* on the basis of its cardioexcitatory activity (Price and Greenberg, 1977), an effect subsequently demonstrated in other mollusk species as well (Painter and Greemberg, 1982; Zatylny-Gaudin and Favrel, 2014). Moreover, in mollusks FMRF-amide-related peptides are involved in regulating gut motility, feeding behavior and reproduction (Dockray, 2004; Zatylny-Gaudin and Favrel, 2014), indicating the functional conservation of this signaling system during evolution.

Little is presently known about the distributions of neurotransmitters and other molecules related to neurotransmission, such as GABA, TH, and ChAT, in the bivalve nervous system. In *Cr. grayanus* GABA-, TH-, and ChAT-LIP neurons together accounted for 12.3% of all immunostaining cells in the CPG, where expression of serotonin and FMRFamide dominated, and 50% of those in the VG, from which serotonin-LIP somata are absent (Figure 8).

γ-aminobutyric acid is an inhibitory neurotransmitter in invertebrates (Sattelle, 1990) and GABA-immunoreactive neurons have been detected in the nervous systems of nudibranch gastropods (Gunaratne et al., 2014), bivalves (Vitellaro-Zuccarello and De Biasi, 1988; Vitellaro-Zuccarello et al., 1991; Cochran et al., 2012) and cephalopods (Cornwell et al., 1993), including the ganglia of the mussels *Mytilus edulis* and *Mytilus galloprovincialis*, the clams *Mercenaria mercenaria* and *Macoma balthica*, and the oyster *Crassostrea virginica* (Vitellaro-Zuccarello et al., 1991; Karhunen et al., 1993; Cochran et al., 2012). In the case of our mussel *Cr. grayanus* GABA-LIP neurons constituted 29.8%, 5.1% and 4%, respectively, of the cells in sections from the CPG and PG (before spawning), whereas numerous GABA-LIP neurons have been detected in the VG of *Mytilus galloprovincialis* (Vitellaro-Zuccarello et al., 1991) and PG of *Macoma balthica* (Karhunen et al., 1993) (after intense spawning). These differences could reflect seasonal variations associated with gonadal development (Mahmud et al., 2014), as well as, possibly, the life-style of the mollusks (free-living versus attached).

Little is presently know about the role of GABAergic neurons in bivalves, with previous investigations simply suggesting that these cells are involved in bilateral coordination and the integration of peripheral motor and sensory information. Our finding here that GABA-LIP neurons are the predominant cell type in the VG is consistent with what and colleagues reported earlier (Cochran et al., 2012).

When applied directly to the gills of the oyster *Crassostrea virginica* and mussel *Mytilus edulis*, GABA exerted no direct effect on cilia activity. However, when serotonin was applied to the cerebral ganglia, treatment with GABA before or afterward blocked stimulation of the beating of the cilia. Moreover, the GABA antagonist, bicuculline methchloride, blocked these effects (Cochran et al., 2012). Inhibition of the effects of serotonin by GABA may be involved in the muscle catch relaxation associated with bivalve adductor contraction.

Choline acetyltransferase (ChAT) has been detected in mollusks by biochemical, electrophysiological and immunohistochemical methods (Bellanger et al., 1998; Perry et al., 1998; Kotsyuba, 2007; Sakaue et al., 2014). Although the distribution of acetylcholine in bivalves has not yet been studied in detail, the clam *Mactra sulcatoria* has huge ChAT-LIP neurons (20–45 μm), of which 13.9% are in the VG, 12.8% in the PG and 8% in the CPG (Kotsyuba, 2007). We also found that in the VG and PG of *Cr. grayanus*, the number of ChAT-LIP neurons is considerable, whereas the CPG contains only solitary neurons of this sort.

Based on comparisons between different bivalves, some investigators propose that development of the ChATergic nervous system is related to the motility of the animal. The dual effects of acetylcholine on muscles have been demonstrated with inhibitors of this neurotransmitter (Kodirov, 2011), which slow the heart beat in *Venus mercenari* (Welsh, 1953), but stimulate both the hearts of all members of the genus *Mytilus* (Welsh, 1961), as well as their smooth muscle (Twarog, 1954) and cilia (Welsh, 1961). The functional role of ChAT-LIP neurons in *Crenomytilus grayanus*, other than participation in catch contraction, remains unknown.

Tyrosine hydroxylase (TH) is a well-recognized marker for neurons containing dopamine, norepinephrine, and epinephrine (i.e., catecholamines, CA). Neurons containing CA have been detected in bivalves, including the mussel *Mytilus edulis* (Stefano and Aiello, 1975), scallops *Patinopecten yessoensis* (Matsutani and Nomura, 1984) and *Placopecten magellanicus* (Pani and Croll, 1995; Smith et al., 1998), *Chlamys farreri* (Kotsyuba, 2009), and clam *Megangulus venulosus* (Kotsyuba, 2011). Despite the similarity of the structure of the nervous systems of bivalves, the number and morphology of their neurons can vary greatly.

Employing both immunostaining for TH and histochemical detection of dopamine (FaGlu), we found neurons expressing these in all *Cr. grayanus* ganglia. The proportion of TH-LIP neurons varies from 7% in the CPG to 14.6–23.2% in the VG and PG. The corresponding proportions for *Megangulus venulosus* vary from 5.5% for the CPG to 7.8% for the VG. The FaGlu technique has also been used to identify dopamine in *Mytilus edulis* and six other bivalve species (Sweeney, 1963). In addition to ganglia, TH-LIP has been detected in the cerebrovisceral connective and branchial nerve.

Direct application of DA to excised gill filaments or the VG or CPG in *Crassostrea virginica* reduced the rate at which the lateral cilia beat in a dose- dependent manner (Carroll and Catapane, 2007). This led to the proposal that the CG contains dopaminergic neurons with synapses in the VG, along with a second set of serotonergic and dopaminergic neurons that innervate the gill. The epithelial cells within the gill on which the lateral cilia are located would have DA receptors that, when activated, decrease the rate of ciliary beating. Thus, at the level of both the CG and VG, DA would inhibit ciliary beating.

Another question we addressed here is whether mollusk neurons are able to proliferate. With the exception of sympathetic neurons, neurons are generally regarded as post-mitotic cells that eventually undergo apoptosis in adult animals, while throughout life glia cells proliferate and replace the neurons that have died (Eccleston et al., 1987). The proliferative cells in mollusks include hemocytes and epithelial cells and, in much lower numbers, the intestine, foot and body wall, and gonads (Marigómez et al., 1999; Hanselmann et al., 2000). The recent detection of PCNA in the brain of adult *Octopus vulgaris* (Bertapelle et al., 2017) has fueled interest in potential neuronal proliferation in mollusks.

Here, we found PCNA-immunopositive neurons in the ganglia of the mussel *Cr. grayanus*. Although the nature of these cells remains unknown, they are not immunostained by any of the antibodies used here, nor do they contain dopamine, so they are probably not neurons. It is highly likely that these PCNA-LIP cells are glia, but without additional information, their definitive identification is not yet possible.

In summary, although the morphology of the ganglionic nervous system of bivalves is relatively simple, a wide variety of neuronal cell types are present. Future elucidation of the involvement of different neurotransmitters in the complex behavior of bivalves is clearly warranted.

## Data Availability Statement

All datasets presented in this study are included in the article/Supplementary Material.

## Author Contributions

All authors had full access to all data in the study and take responsibility for its integrity and the accuracy of the analysis. EK and VD: study concept and design. EK: material collection and preparation. AK: FaGlu experiments. VD and PK: immunohistochemistry. VD and EK: data analysis and interpretation. VD: study supervision.

## Conflict of Interest

The authors declare that the research was conducted in the absence of any commercial or financial relationships that could be construed as a potential conflict of interest.
