# Supplementary figures and images for: Distribution of Molecules Related to Neurotransmission in the Nervous System of the Mussel Crenomytilus grayanus
Source: Front Neuroanat. 2020 Jun 30;14:35. doi: 10.3389/fnana.2020.00035 (PMC7344229; doi:10.3389/fnana.2020.00035)

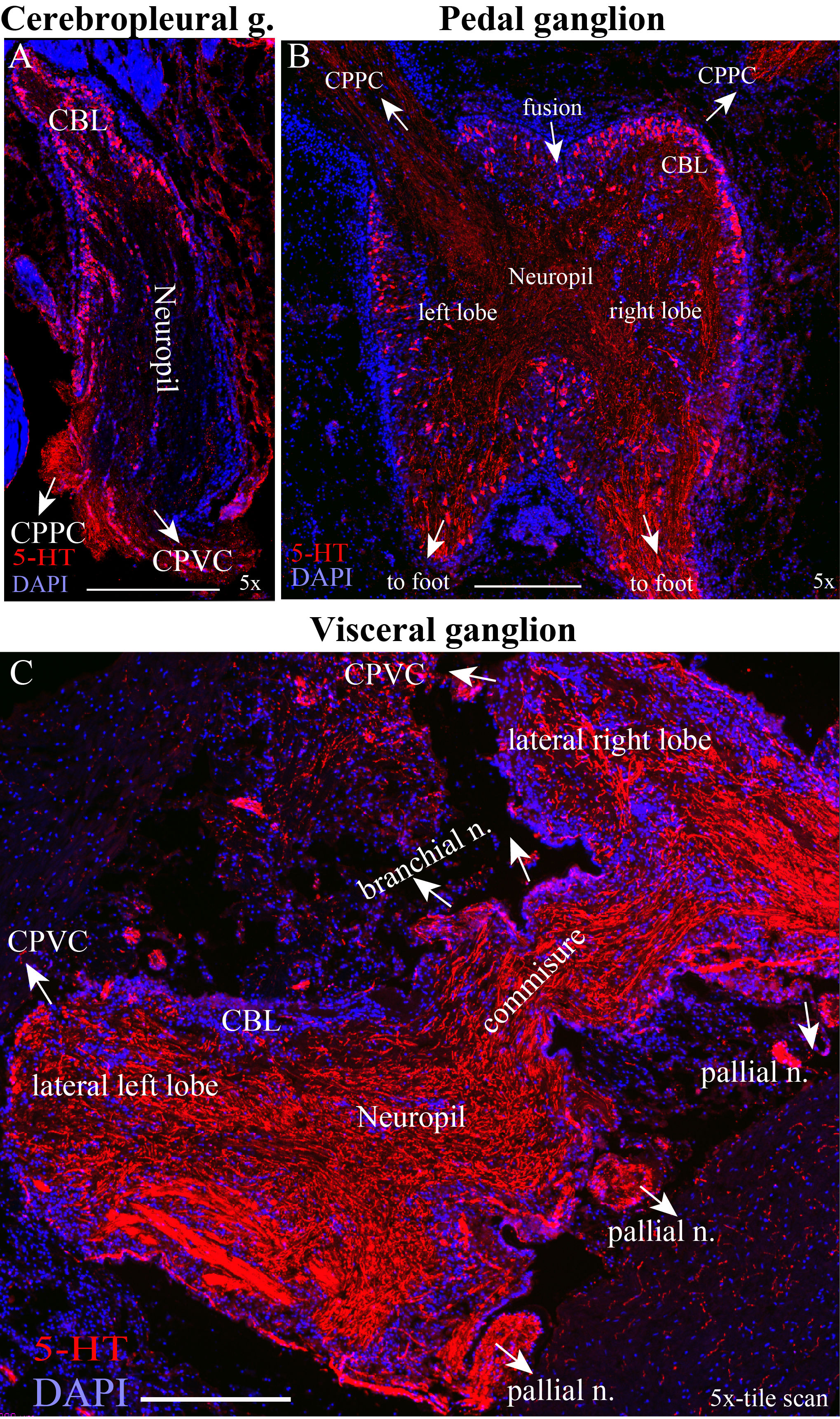

Supplement: Supplementary file 1 [file Image_1.jpg]
