# Supplementary material for: Distribution of Molecules Related to Neurotransmission in the Nervous System of the Mussel Crenomytilus grayanus
Source: Front Neuroanat. 2020 Jun 30;14:35. doi: 10.3389/fnana.2020.00035 (PMC7344229; doi:10.3389/fnana.2020.00035)
Supplement: Supplementary file 2 [file Image_1.pdf]

Supplementary information

Distribution of molecules related to neurotransmission in the nervous system of the mussel *Crenomytilus grayanus*

Elena Kotsyuba[1], Alexander Kalachev[1], Polina Kameneva[1]& Vyacheslav Dyachuk[1,2*]

[1] A.V. Zhirmunsky National Scientific Center of Marine Biology, Far Eastern Branch, Russian Academy of Sciences, Vladivostok 690041, Russia
[2]Department of Nanophotonics and Metamaterials, IFMO University, St. Petersburg, Russia

***Corresponding author:** slavad83@gmail.com

Supplementary Figure 1. Whole structure of the main ganglia of the mussel *Crenomytilus grayanus.* (**a**) cerebropleural ganglion; (**b**) pedal ganglion; (**c**) visceral ganglion stained for 5-HT with DAPI. CPG, cerebropleural ganglion; PG, pedal ganglion; VG, visceral ganglion; CPPC, cerebral-pleural-pedal connectives; CPVC, cerebral-pleural-visceral connectives. Bar, 1mm.

Other Supplementary Information for this manuscript includes the following:

Supplementary Movie 1

Whole-mount immunostaining of the cerebropleural ganglion with antibody against 5-HT.

Supplementary Movie 2

Whole-mount immunostaining of the cerebropleural ganglion with antibody against FMRFamide.

Supplementary Movie 3

Whole-mount immunostaining of the pedal ganglion with antibody against 5-HT.

Supplementary Movie 4

Whole-mount immunostaining of the pedal ganglion with antibody against FMRFamide.

Supplementary Movie 5

Whole-mount immunostaining of the visceral ganglion with antibody against FMRFamide.
